# Editorial: Advanced Non-invasive Photonic Methods for Functional Monitoring of Haemodynamics and Vasomotor Regulation in Health and Diseases

**DOI:** 10.3389/fphys.2020.00325

**Published:** 2020-04-15

**Authors:** Alexey Goltsov, Viktor V. Sidorov, Sergei G. Sokolovski, Edik U. Rafailov

**Affiliations:** ^1^Institute of Cybernetics, Russian Technological University, Moscow, Russia; ^2^M&S Decisions LLD, Moscow, Russia; ^3^SPE “LAZMA” Ltd., Moscow, Russia; ^4^Optoelectronics and Biomedical Photonics Group, Aston Institute of Photonic Technologies, Aston University, Birmingham, United Kingdom

**Keywords:** non-invasive multimodal photonic diagnostics, laser Doppler flowmetry, NIRS, optoacoustic technique, ischemic stroke, hemodynamics regulation

In the present Research Topic we collected papers devoted to the different aspects of the non-invasive photonic techniques capable monitoring real-time microvascular hemodynamics in health and pathology. Compact laser instruments for near-infrared spectroscopy (NIRS), Laser Doppler Flowmetry (LDF), diffuse correlation spectroscopy (DCS), NIRS flow oximetry, and others are routinely and widely used for concurrent non-invasive measurement of different parameters of hemocirculation such as blood perfusion, mean arterial pressure, oxy- and deoxyhemoglobin concentrations, and others. Further development and introduction of these approaches and systems to clinical practice will lead to enrichment and step-by-step substitution of laboratory tests with enabling bed-side diagnostics and wireless distantly controlled patient condition monitoring.

The biophotonic approaches became a powerful tool to study dynamic processes such as vascular tone autoregulation and functional hyperaemia which are controlled by vessel vasculature rhythms derived from different regulatory systems: nervous sympathetic and parasympathetic, hormonal and mechanical muscle regulation. A promising area for research is establishing an autoregulatory link between blood flow and level of the metabolic activity where combined LDF and fluorescent approaches can bring significant advantages in the diagnostics. Spectral analysis of different optical data arrays can provide valuable information on neuronal-vasculature and muscular-vasculature coupling autoregulation mechanism under intense stress and/or physical activity in health and at various pathologies. Due to the morphological and physiological differences in the microcirculation bed of the human skin, a non-invasive detailed research of blood microcirculation can be organized using a new class of wearable wireless laser flowmeters adopted to the range of the research areas.

This Research Topic brings together the cutting-edge applications of non-invasive biophotonic approaches and techniques allowing to translate personalized data array analysis to distinguished diagnosis.

In the research paper, Goltsov et al. applied spectral analysis of the LDF signals for the investigation of cerebrovascular hemodynamics in patients with post-acute ischemic stroke. The observed asymmetry in LDF signals measured on the stroke-affected and unaffected hemispheres of the patients was analyzed by computational modeling of vasomotion of arteriole diameter.

Zherebtsova et al. discussed the development and application of the optical non-invasive diagnostic approach for the detection and evaluation of the microcirculatory severity and metabolic disorders in patients with rheumatic diseases and diabetes mellitus. The proposed methods include the joint use of LDF, absorption spectroscopy and fluorescence spectroscopy in combination with functional tests in the field of rheumatology and endocrinology.

Semyachkina-Glushkovskaya et al. investigated brain hemorrhages in newborn rats using an integrative approach including magnetic resonance imaging, monitoring of cerebral blood flow using Doppler coherent tomography, oximetry, coherent-domain optical technologies for visualization of the cerebral blood flow, investigations of the deformability of red blood cells, and morphological analysis of brain tissues. Combination of these techniques in the monitoring of the pre- and post-hemorrhage periods allowed authors to investigate a time-dependent stress inducing changes in neonatal brain areas which are involved in higher cognitive functions in progress of the pathology.

In the method article, Caicedo et al. developed a new computational method for the decomposition of NIRS signals into the partial linear contributions of different physiological signals. Authors combined Oblique Subspace Projections in order to define adequate signal subspaces and the linear regression model of NIRS signals to calculate regression parameters and estimate the contribution of various input variables. Authors evaluate the performance of the developed methods by case studies in the field of cerebral hemodynamics monitoring using NIRS and concomitant measurements of MABP, EtCO2, HR, SaO2, and ECMO signals.

In mini-survey, Esenaliev reviewed optoacoustic technique which is a novel medical diagnostic platform for non-invasive measurement of physiologic variables, functional imaging, and hemodynamics monitoring. Authors discussed the application of optoacoustic methods to the measurement of important physiologic parameters including temperature, thermal coagulation, freezing, concentration of molecular dyes and nanoparticles in organs, oxygenation, and hemoglobin concentration with high temporal and spatial resolution.

Guo et al. analyse the daily rhythm of dynamic cerebral autoregulation by simultaneous measurement of the arterial blood pressure at the brachial artery and cerebral blood flow velocity over the course of a day in healthy adults.

Obata et al. used simultaneously recorded the ECG and plethysmograph in the investigation of the effect of body position and exercise on pulse arrival time taken for the pulse wave to reach the vasculature of various peripheral tissue beds. Authors analyzed vascular autoregulation mechanisms that maintain the optimal arrival time irrespective of its distance from the heart.

Several papers in the Research Topic are devoted to the biophysical investigation of vascular regulation at the molecular, cellular, and tissue levels. Revin et al. presented results of the detailed biophysical investigation on the hyperglycaemia effect on morphological changes in erythrocytes. Authors combined Raman spectroscopy in the observation of conformational changes in hemoglobin with laser interference microscopy to determine structural transition and a composition change in the erythrocyte membranes at graduated hyperglycaemia. Ivanov et al. investigated the possibility of the application of microwave radiometry method to non-invasive monitoring of the physicochemical properties of circulating proteins. In brief research report article, authors provided results on the application of commercially available microwave radiometry equipment to the investigation of denaturation kinetics of albumin in solution. Plekhanova et al. investigated the potential role of plasminogen activators (PA) in the promotion of outward arterial remodeling after injury in rat common carotids. Authors studied a gene expression profile change after injury and local treatment with the recombinant PA in the vessel wall related to vascular tone. Li et al. discussed the prospective researches of the plasma matrix metalloproteinase-9 (MMP-9) contents that can serve as a predictive indicator for cardiovascular disease mortality in the Chinese Han population.

The results presented in this Research Topic by different research groups show a wide spectrum of the current directions for the development and application of biphotonic techniques in medicine. As seen, the mainstream research in biophotonics focuses on the monitoring and processing of concurrently collected biomedical signals in patients to enhance current clinical diagnosis and develop novel diagnostic techniques and equipment.

## Author Contributions

All authors listed have made a substantial, direct and intellectual contribution to the work, and approved it for publication.

### Conflict of Interest

AG is employed by company M&S Decisions LLD. VS is employed by the company SPE “LAZMA” Ltd. The remaining authors declare that the research was conducted in the absence of any commercial or financial relationships that could be construed as a potential conflict of interest.

